# Numerical Study of the Effect of the Port Angle of the Superior Vena Cava Supplying Cannula on Hemodynamics in the Right Atrium in VV-ECMO

**DOI:** 10.3390/biomedicines12102198

**Published:** 2024-09-26

**Authors:** Xinrui Ma, Kaihang Xu, Bin Gao

**Affiliations:** College of Chemistry and Life Science, Beijing University of Technology, Beijing 100124, China; mxr@bjut.edu.cn (X.M.);

**Keywords:** VV-ECMO, superior vena cava supplying, right atrium, hemodynamics, cannula

## Abstract

**Objective:** To elucidate the pattern of the influence of the port angle of the superior vena cava supplying cannula (SVCS) on hemodynamics within the right atrium in VV-ECMO. **Methods:** A three-dimensional model of the right atrium was established based on CT images of a real patient. The 3D models of the SVCS and inferior vena cava draining cannula (IVCD) were established based on the Edwards 18Fr and Medos 22Fr real intubation models, respectively. Based on these models, three-dimensional models of the SVCS ports with bending angles of −90°, −60°, −30°, 0°, 30°, 60°, and 90° in the plane formed by the centerline of the SVCS and the center point of the tricuspid valve (TV) were established. Transient-state computational fluid dynamics (CFD) was performed to clarify the right atrium blood flow pattern and hemodynamic states at different SVCS port orientation angles. The velocity clouds, wall pressure, wall shear stress (WSS), relative residence time (RRT), and recirculation fraction (RF) were calculated to assess hemodynamic changes in the right atrium at different angles of the port of the SVCS. **Results:** As the angle of the port of the superior chamber cannula changed, the location of the high-velocity blood impingement from the SVCS changed, and the pattern of blood flow within the right atrium was dramatically altered. The results for the maximum right atrial wall pressure were 13,472 pa, 13,424 pa, 10,915 pa, 7680.2 pa, 5890.3 pa, 5597.6 pa, and 7883.5 pa (−90° vs. −60° vs. −30° vs. 0° vs. 30° vs. 60° vs. 90°), and the results for the mean right atrial wall pressure were 6788.9 pa, 8615.1 pa, 8684.9 pa, 6717.2 pa, 5429.2 pa, 5455.6 pa, and 7117.8 pa ( −90° vs. −60° vs. −30° vs. 0° vs. 30° vs. 60° vs. 90°). The results of the maximum right atrial wall WSS in the seven cases were 63.572 pa, 55.839 pa, 31.705 pa, 39.531 pa, 40.11 pa, 28.474 pa, and 35.424 (−90° vs. −60° vs. −30° vs. 0° vs. 30° vs. 60° vs. 90°), respectively, and the results of the mean right atrial wall WSS results were 3.8589 pa, 3.6706 pa, 3.3013 pa, 3.2487 pa, 2.3995 pa, 1.3304 pa, and 2.0747 pa (−90° vs. −60° vs. −30° vs. 0° vs. 30° vs. 60° vs. 90°), respectively. The results for the area percentage of high RRT in the seven cases were 3.44%, 2.23%, 4.24%, 1.83%, 3.69%, 7.73%, and 3.68% (−90° vs. −60° vs. −30° vs. 0° vs. 30° vs. 60° vs. 90°), and the results for the RF were 21.57%, 23.24%, 19.78%, 12.57%, 10.24%, 5.07%, and 8.05% (−90° vs. −60° vs. −30° vs. 0° vs. 30° vs. 60° vs. 90°). **Conclusions:** The more the port of the SVCS is oriented toward the TV, the more favorable it is for reducing RF and the impingement of blood flow in the right atrial wall, but there may be an increased risk of RRT. The opposite orientation of the SVCS port to the TV is not conducive to reducing flow impingement on the right atrial wall and RF.

## 1. Introduction

Venovenous extracorporeal membrane oxygenation (VV-ECMO) is an extracorporeal life-support technique, which mainly consists of the superior vena cava supplying cannula (SVCS), inferior vena cava draining cannula (IVCD), centrifugal pump, and oxygenator. The centrifugal pump pumps venous blood through the IVCD to the oxygenator, which increases the oxygen content of the blood through the oxygenator, and then delivers the blood back to the right atrium through the SVCS in order to increase the oxygen content of the patient’s circulation [1], removing excess CO_2_, assisting in tissue perfusion and metabolism [2], and thereby increasing the oxygen content of the patient’s circulation. It has gradually become a major research focus in the treatment of respiratory failure [3] and a new therapeutic means [4].

With the widespread clinical application of VV-ECMO, some of its issues have also begun to emerge, such as recirculation, blood damage, and thrombus formation [5], which are frequent complications. Improper positioning of the SVCS and the IVCD can lead to abnormal hemodynamic characteristics, causing the various aforementioned complications [6,7]. Research has found that by adjusting factors such as the type of cannula, the position of the cannula, and the internal diameter of the cannula, the blood flow state within the right atrium can be altered to achieve the purpose of reducing complications. Bonacchi et al. [8] observed clinically that adjusting the position and angle of the cannula can significantly affect the efficiency of oxygenation, and changing the angle of the SVCS has a positive effect on reducing recirculation. Xie et al. [9] found that the position and angle of the ECMO cannula have an important impact on the hemodynamics of the right atrium, and the method of simulating a circulatory circuit [10] confirmed that different cannula positions in VV-ECMO have a direct impact on the hemodynamics of the circulatory system, especially recirculation. Steven et al. [11] used the fluid–structure interaction (FSI) method to analyze the recirculation effect in the right atrium of VV-ECMO under various cannula parameters, such as the position and internal diameter of the cannula, and found that various cannula parameters can cause changes in recirculation. The aforementioned studies indicate that cannula parameters play a significant role in reducing recirculation and complications by altering the blood flow state within the right atrium. However, the specific impact of the angle of the port of the SVCS on the hemodynamics within the right atrium is still unclear, posing difficulties for clinical treatment.

To address these issues, this study established three-dimensional models with seven different angles for the SVCS port. Computational fluid dynamics (CFD) methods were used to perform transient fluid–structure interaction (FSI) calculations with the models. Parameters such as velocity field, volume of low-velocity areas, relative residence time (RRT), and recirculation rate (RF) were extracted to assess the blood flow state within the right atrium under different angles of the SVCS port. The aim was to provide a theoretical reference for the cannulation parameters used in the clinical treatment of patients with V-V ECMO.

## 2. Method

### 2.1. 3D Geometry Model Reconstruction

**Figure 1:**
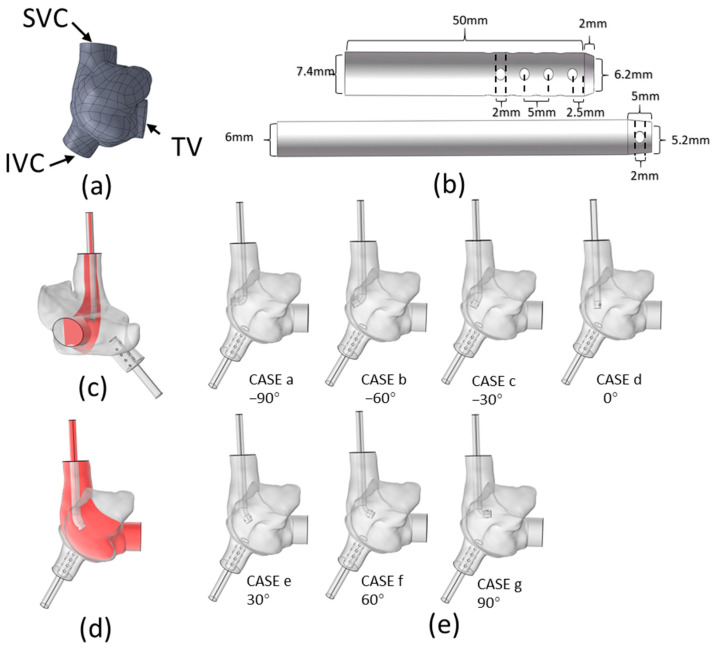
Experimental Model: (**a**) Right atrial model; (**b**) SVCS model (bottom) and IVCD model (top); (**c**,**d**) Different angle displays of the plane where the cannula bends; (**e**) Assembly models with seven different angles of the SVCS port.

MRI image data of a heart from a patient with respiratory failure at Wuhan Asian Heart Hospital were acquired for the purpose of three-dimensional reconstruction of a right atrial model. This type of atrium is considered a commonly encountered shape in clinical practice by clinicians. Initially, the images were imported and a preliminary reconstruction was conducted using Mimics software (version 20.0). Next, the Freeform Plus 2017 (by Geomagic, Cary, NC, USA) was employed to refine the model by smoothing, yielding a geometric model of the right atrium, as illustrated in Figure 1a. A genuine internal jugular vein perfusion catheter (Edwards, Irvine, CA, USA, 18Fr) was selected to serve as the SVCS and a femoral vein drainage catheter (Medos, Beijing, China, 22Fr) as the IVCD. Based on these, the geometric models of the SVCS and IVCD were established, as shown in Figure 1b. SolidWorks 2018 was utilized to process the port of the SVCS, using the plane formed by the centerline of the SVCS and the midpoint of the TV (Figure 1c,d shows different angles of this plane) to establish geometric models of the SVCS port facing in various directions. The angle facing towards the TV is defined as positive. A schematic diagram of the assembled model is shown in Figure 1e.

### 2.2. Meshing Generation

This study employs COMSOL to generate high-quality free tetrahedral meshes. To determine the appropriate mesh size, a three-dimensional model with a 30° SVCS bending angle was selected, and three mesh quantities, of 0.22 million, 0.43 million, and 0.76 million, were set. This study selects four characteristic moments of the right atrial wall average pressure as the standard for mesh independence testing: the moment when the tricuspid valve closes, T1 (0.3 s), the maximum peak during the tricuspid valve opening, T2 (0.545 s), the lowest valley, T3 (0.67 s), and the second largest peak of the opening, T4 (0.755 s). The mesh size is considered to meet the requirements of this study when the relative error is less than 5%. The results indicate that once the mesh quantity reaches 0.43 million, the relative errors of the average pressure on the right atrial wall at the four characteristic moments are 1.06%, 1.85%, 1.11%, and 0.53%, all of which are less than 5%, meeting the criteria. Three boundary layers are generated to analyze the flow field in the inner wall of the right atrium, with the mesh count for each scenario listed in Table 1.

### 2.3. Boundary Conditions

**Figure 2:**
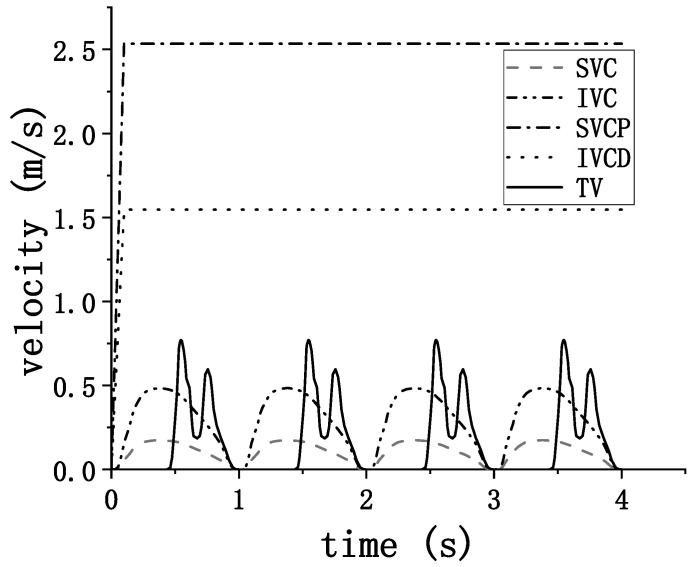
Boundary Conditions at Each Inlet and Outlet.

This computational model features a total of five inlets and outlets, which include the superior vena cava (SVC) inlet, the inferior vena cava (IVC) inlet, and the SVCS inlet as the entry points. The exit points consist of the IVCD outlet and the TV outlet. Velocity boundary conditions are applied to all inlets and outlets. The velocity boundary conditions for the SVC inlet, inferior vena cava (IVC) inlet, and tricuspid valve outlet are based on the research by Steven [11], in which a patient’s flow curve over time was obtained from an echocardiographic study, including both the passive and active phases of right atrial emptying. In accordance with clinical practice, the total flow rate at the inlets and outlets is set to 4 L/min, with the ratio of the return blood volume of the SVC to IVC being 0.32:0.68. The flow rate at both the SVCP inlet and the IVCD outlet is set to 3 L/min, with a ramp transition added during the initial 0.1 s to improve computational stability. These are then converted into velocity boundary conditions based on the areas of the inlets and outlets of the model. The velocity–time curves for the five inlets and outlets are shown in Figure 2.

### 2.4. Calculation Settings

Some previous studies have indicated that it is acceptable to model blood [5,12] and the atrial wall [11] as linear materials in VV-ECMO. To simplify the calculations, we modeled blood and the atrial wall as linear materials. In this study, the right atrial wall is modeled as a linear elastic material with a density of 1060 kg/m^3^ [13], a Young’s modulus of 0.2 MPa [14], and a Poisson’s ratio of 0.46 [15]; the cannula is set as a rigid material. Blood is defined as an isotropic, incompressible Newtonian fluid with a density of 1050 kg/m³ and a viscosity of 0.0035 Pa·s. The inner surface of the right atrial wall is subjected to a no-slip boundary condition. The boundaries of the superior vena cava and inferior vena cava walls within the right atrial wall model, as well as at the tricuspid annulus, are set with fixed constraints. The contact surface between the fluid and the right atrial wall is set as a fluid–structure interaction interface. The cardiac cycle is set to 1 s, and a fully coupled solver is used for the solution, with a convergence criterion set to 10^−3^. To quantify the recirculation rate, the particle tracking module in COMSOL Multiphysics 6.2 is utilized. Over the 0–1 s interval, 500 particles are released every 0.1 s, for a total of 5000 particles released at the SVCS inlet. The right atrial wall is set to reflect particles, and particle freezing is implemented at the TV and the IVCD outlet. Particle counters are added at these locations. The calculations are performed over four cardiac cycles to ensure that more than 90% of the particles exit through the outlet boundary. The MUMPS direct solver is employed for the particle tracking calculations.

### 2.5. Hemodynamic Analysis

The impact of different cannula positions on the hemodynamics within the right atrium is assessed using parameters such as the blood flow velocity cloud map, right atrial wall pressure cloud map, right atrial wall shear stress (WSS) cloud map, recirculation fraction (RF), and relative residence time (RRT). RRT refers to the time required for particles to flow through a certain fluid region [16]. Recirculation refers to the phenomenon whereby a portion of the oxygenated blood entering the right atrium through the SVCS does not enter the systemic circulation but is instead directly drawn out by the IVCD [17]. The RRT was defined using Equation (1):

(1) $$RRT=1/[\left(1-2OSI\right)TAWSS]$$

where RRT stands for relative residence time, OSI stands for oscillatory shear index, and TAWSS stands for time-averaged wall shear stress. The OSI and TAWSS were defined using Equations (2) and (3):

(2) $$OSI=0.5\times \left(1-\frac{\left|\int_{0}^{T}\tau_{w}dt\right|}{\int_{0}^{T}\left|\tau_{w}\right|dt}\right)$$

(3) $$TAWSS=\frac{1}{T}\int_{O}^{T}\left|\tau_{w}\right|dt$$

where $\tau_{w}$ represents the wall shear stress; *T* represents the duration of one cardiac cycle.

The RF was defined using Equation (4):

(4) $$RF\left(\%\right)=\frac{M_{IVCD}}{M_{IVCD}+M_{TV}}\times 100\%$$

where $M_{IVCD}$ represents the number of particles frozen at the IVCD; $M_{TV}$ represents the number of particles frozen at the TV.

## 3. Results

### 3.1. Right Atrial Blood Flow Field

**Figure 3:**
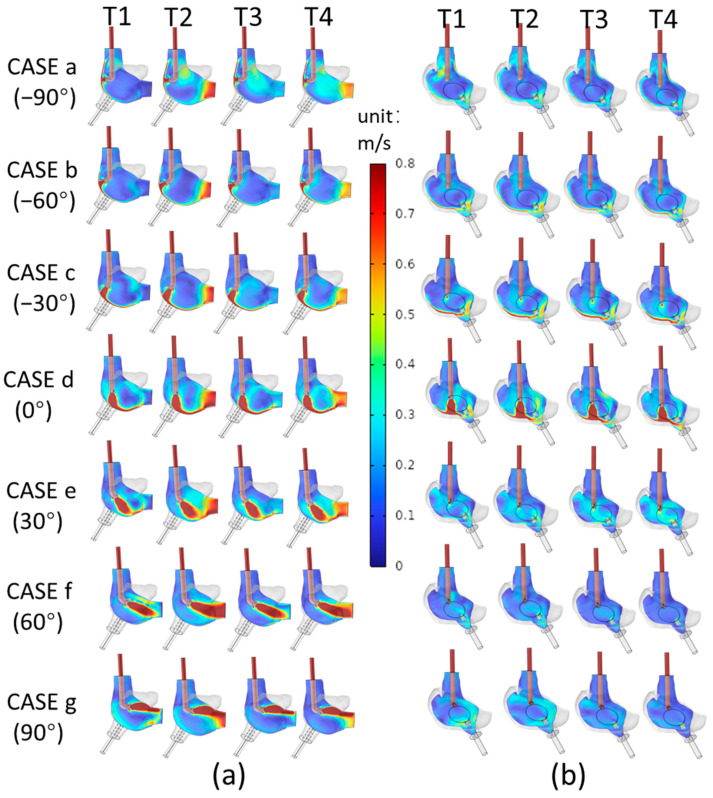
Velocity Cloud Map Results. (**a**) Velocity cloud map on the plane formed by the midline of the SVCS and the midpoint of the TV; (**b**) Velocity cloud map on the plane formed by the midline of the SVCS and the midpoint of the IVCD port.

Figure 3a displays the velocity cloud map of the plane formed by the centerline of the SVCS and the midpoint of the TV, while Figure 3b shows the velocity cloud map of the plane formed by the centerline of the SVCS and the midpoint of the port of the IVCD. From Figure 3a, it can be seen that at the four characteristic moments, for CASE a (−90°), CASE b (−60°), and CASE c (−30°), the blood flow from the SVCS directly hits the right atrial wall far from the TV, with only a small amount of blood flowing through the lower part of the right atrial wall to the TV area. In CASE d (0°), the blood flow from the SVCS directly impacts the lower part of the right atrial wall, with some high-velocity blood following the curve of the lower right atrium into the entrance of the TV. For CASE e (30°), CASE f (60°), and CASE g (90°), the high-velocity blood flow from the SVCS directly hits the right atrial wall close to the TV, with CASE f (60°) showing the high-velocity blood flow directly impacting the TV position. In these three cases, a large amount of high-velocity blood flows into the TV. From Figure 3b, it can be observed that during the positive angle orientation of the SVCS, as the angle increases from 0° to 60°, the velocity at the port of the IVCD continuously decreases. However, when the angle is increased from 60° to 90°, there is a slight increase in the velocity at the port of the IVCD.

### 3.2. Right Atrial Wall Pressure

**Figure 4:**
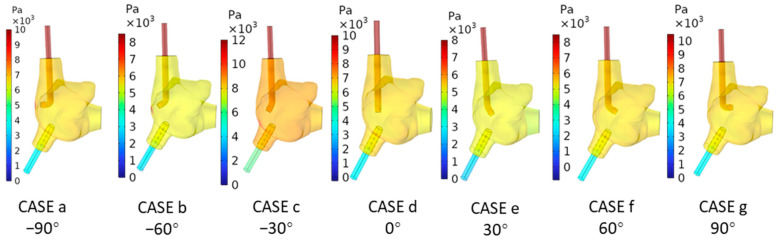
Pressure Cloud Maps on the Right Atrial Wall at T2 under Seven Conditions.

Figure 4 displays the pressure cloud maps of the right atrial wall at T2 under seven different angles of the SVCS, while Table 2 presents the statistical analysis of the right atrial wall pressure. From Figure 4, it can be observed that all cases exhibit relatively high-pressure areas on the right atrial wall in the region facing the SVCS. According to Table 2, when the SVCS angle is at −90°, the maximum pressure on the right atrial wall is the highest, reaching 13,472 Pa. As the angle of the SVCS increases from −90° to 60°, the maximum pressure on the right atrial wall continuously decreases, with the minimum maximum pressure occurring in CASE f (60°), at 5597.6 Pa. When the angle of the SVCS reaches 90°, the maximum pressure on the right atrial wall increases slightly to 7883.5 Pa. From the average pressure on the right atrial wall, it is evident that CASE c (−30°) has the highest average wall pressure, which is 8684.9 Pa. CASE e (30°) has the lowest average wall pressure, which is 5429.2 Pa.

### 3.3. Right Atrial Wall WSS

**Figure 5:**
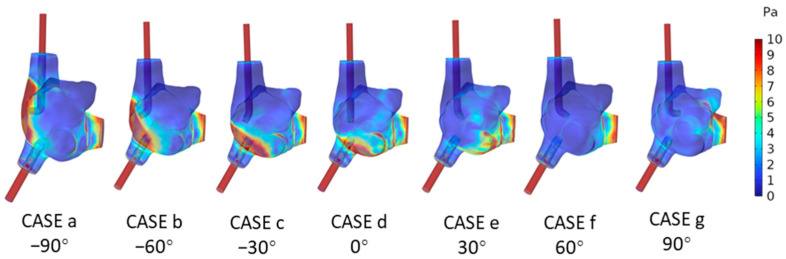
WSS Cloud Maps on the Surface of the Right Atrium at T2 under Seven Conditions.

Figure 5 displays the WSS cloud maps of the right atrial wall at T2 under seven different angles of the SVCS, while Table 2 presents the statistical analysis of the right atrial wall WSS. In Figure 5, it can be observed that in all cases, the region of the right atrial wall directly facing the SVCS exhibits areas of high WSS. To quantify the WSS on the right atrial wall under various conditions, we extracted the maximum and average WSS values of the right atrial wall at time T2 for the seven cases, as shown in Table 2. The maximum WSS value decreases as the angle of the SVCS port increases from −90° to 60°. CASE a (−90°) has the highest maximum WSS value, at 63.57 Pa, while CASE f (60°) has the lowest, at 28.474 Pa. The maximum WSS value for CASE g (90°) shows a slight increase compared to CASE f (60°), reaching 35.424 Pa. The pattern of change in the average WSS is consistent with that of the maximum WSS, with the highest value occurring in CASE a (−90°), at 3.8589 Pa, and the lowest in CASE f (60°), at 1.3304 Pa.

### 3.4. Recirculation Fraction

The magnitude of the recirculation rate was calculated using the particle counter function in COMSOL, by counting the number of particles at the TV and the IVCD outlet to determine the size of the recirculation rate. The smaller the recirculation rate, the better the oxygenation effect. The statistical results are shown in Table 3, and the RF was calculated using Equation (4). According to Table 3, starting from CASE b (−60°), as the cannula angle increases, the RF continuously decreases. When the angle of the SVCS port reaches 60°, the recirculation rate is at its minimum, at 10.88%. At this point, the port of the WVCS is facing towards the TV. When the cannula port angle continues to increase, the recirculation rate becomes higher.

### 3.5. Relative Residence Time

**Figure 6:**
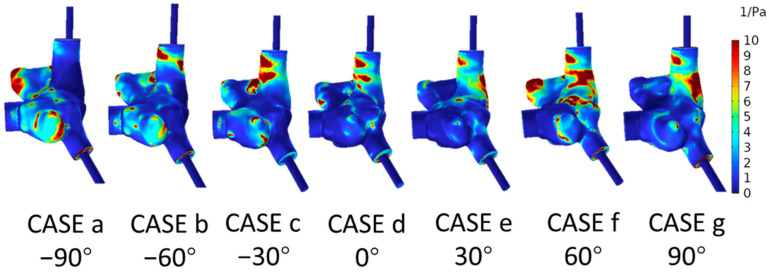
RRT Cloud Maps on the Surface of the Right Atrium under Seven Conditions.

The RRT reflects the stagnation velocity of the fluid. A lower RRT value indicates that the degree of blood flow stagnation within the right atrial fluid region is lower; conversely, a higher RRT value indicates a higher degree of blood flow stagnation in the fluid region. When the RRT exceeds 10 Pa^−1^, the risk of thrombus formation within the fluid increases [18]. Figure 6 illustrates the distribution of the RRT within the right atrium under seven different angles of the superior vena cava cannula port. As can be seen from the figure, the RRT distribution varies among the seven scenarios: In CASE a (−90°), high-RRT areas are primarily located at the tip of the right atrial appendage and the protrusion near the TV. For CASE b (−60°) and CASE c (−30°), the high-RRT areas are mainly distributed near the entrance of the SVC and the protrusion of the right atrium close to the TV. In CASE c (−30°), the high-RRT area at the SVC extends to the junction of the SVC and the right atrial appendage. In CASE d (0°), the high-RRT areas are predominantly concentrated near the SVC. CASE e (30°) shows high-RRT areas at the junction of the SVC and the right atrium. CASE f (60°) exhibits the largest and most dispersed high-RRT areas, with the most severe regions being the tip of the right atrial appendage, the SVC, and the junction with the right atrium. CASE g (90°) presents a large area of high RRT at the junction of the SVC and the right atrium, which is far from the TV. The simulation results of the proportion of high-RRT areas under the seven conditions are shown in Table 2. High-RRT areas occurred in all seven conditions, with CASE d (0°) having the smallest proportion of high-RRT areas, at 1.83%, and CASE f (60°) having the largest proportion, at 7.73%.

## 4. Discussion

The angle of the SVCS port is an important factor affecting the therapeutic effect of VV-ECMO, closely related to the hemodynamic parameters and the degree of recirculation with VV-ECMO assistance. Previous studies have explored the angles of the upper and lower cannula ports in VV-ECMO to some extent, but mostly using clinical observation or experimental methods, and the division of the angles was not fine enough to clarify the impact of different angles of the SVCS port on the hemodynamics within the right atrium. This study applies computational fluid dynamics (CFD) to the context of VV-ECMO to observe the impact on hemodynamics under extracorporeal circulatory support. By adjusting the angle at the SVCS port, three-dimensional models with different angles were obtained and calculated. Parameters such as the right atrial velocity cloud map, right atrial wall pressure, right atrial wall WSS, right atrial wall RRT, and RF were extracted to study the impact of different SVCS port angles on the hemodynamics within the right atrium.

Previous studies have indicated that high-velocity blood flow impact resulting in localized high pressure and WSS is a significant hemodynamic indicator [19,20]. Sustained local high pressure and high WSS may lead to damage to the right atrial wall. The results from this study show that the locations of high-velocity blood flow impact revealed by the velocity cloud map generally correspond to the regions of high pressure and high WSS displayed by the pressure and WSS maps, suggesting that the high-velocity blood ejected from the SVCS port may impact the nearby right atrial wall, leading to areas of high pressure and high WSS in that region. Among the seven cases in this study, the minimum values of both the maximum and the average right atrial wall pressure occurred in CASE f (60°), and the minimum values of both the maximum and the average WSS were also found in CASE f (60°). Therefore, to minimize the impact of blood flow on the right atrial wall, setting the angle of the SVCS port to 60° may be a more suitable option.

In VV-ECMO, recirculation is considered a significant factor that reduces the efficiency of oxygen transport [18]. Some researchers have investigated the factors affecting recirculation in VV-ECMO. Konomi [6] used an animal experiment to fix the IVCD in the inferior vena cava and move the SVCS position up and down, finding that the farther the SVCS cannula position is from the IVCD cannula, the lower the RF tends to be. Steven [11] used a fluid–structure interaction method to roughly observe the impact of the angle of the SVCS on RF and found that when the SVCS is oriented towards the TV, the RF is significantly reduced.

The results of this study are consistent with Steven’s findings. When the SVCS port is oriented towards the TV, the RF is minimized. When the SVCS port is oriented in the opposite direction to the TV, the RF rises significantly. This suggests that orienting the SVCS port more towards the TV is more conducive to reducing recirculation.

The RRT reflects the stagnation velocity of the fluid, and when the RRT exceeds 10 Pa⁻¹, the risk of thrombus formation within the fluid increases [18]. According to the results of this study, the smallest proportion of high-RRT areas among the seven scenarios occurred in CASE d (0°), while the largest proportion was observed in CASE f (60°). This implies that there is a higher likelihood of thrombus formation under the conditions of CASE f (60°). The reason for this might be that when the SVCS port is oriented towards the TV, a significant amount of blood flows directly out through the TV, leading to insufficient blood flow velocity on the opposite side of the TV at the right atrial wall and the right atrial appendage, resulting in a larger area of high RRT. Therefore, even though CASE f (60°) has the smallest impact of blood flow on the right atrial wall and the lowest RF, considering the RRT, it poses the greatest risk of thrombus formation. Despite the use of heparin during surgery to delay thrombus generation, the issue of thrombus formation must still be considered when using VV-ECMO for extended periods. It is suggested that in actual clinical surgical procedures, a comprehensive consideration of various hemodynamic parameters is necessary, and the appropriate orientation angle of the SVCS port should be selected based on the actual situation to reduce complications.

This study also has certain limitations. Firstly, the patient sample size is insufficient; in the future, it will be necessary to increase the number of samples to obtain more universally applicable results. Secondly, complications of VV-ECMO, in addition to thrombus formation and recirculation, may also include renal dysfunction, neurological damage, etc. [21], which could potentially affect the effectiveness of extracorporeal life support. Future research could extend to more aspects, especially integrating multiple factors for study, to provide clinical data support.

## 5. Conclusions

The orientation of the SVCS port plays a critical role in hemodynamics during V-V ECMO. The orientation of the cannula port towards the TV tends to reduce the impact of blood flow on the right atrial wall and the RF. However, overly aligning the cannula port towards the TV could potentially increase the risk of thrombus formation. Conversely, when the SVCS port is oriented away from the TV, towards the opposite side, both the impact on the right atrial wall and the RF are likely to increase. In clinical practice, it is recommended to take a comprehensive approach by considering various hemodynamic parameters to minimize the side effects of V-V ECMO.

## Figures and Tables

**Table 1:** Mesh Independency Test Results.

	T1	T2	T3	T4
0.22 million	2270.8	5176.3	2341.4	879.3
relative error	1.94%	4.66%	6.83%	9.86%
0.43 million	2315.8	5429.2	2513.1	975.43
relative error	1.06%	1.85%	1.11%	0.53%
0.76 million	2410.7	5681.6	2611.3	1018.6

**Table 2:** Statistics of Maximum Pressure, Average Pressure, Maximum WSS, Average WSS, and Proportion of High-RRT Area on the Right Atrial Wall at T2 under Seven Conditions.

	CASE a −90°	CASE b −60°	CASE c −30°	CASE d 0°	CASE e 30°	CASE f 60°	CASE g 90°
Maximum Pressure (Pa)	13,472	13,424	10,915	7680.2	5890.3	5597.6	7883.5
Average Pressure (Pa)	6788.9	8615.1	8684.9	6717.2	5429.2	5455.6	7117.8
Maximum WSS (Pa)	63.572	55.839	31.705	39.531	40.11	28.474	35.424
Average WSS (Pa)	3.8589	3.6706	3.3013	3.2487	2.3995	1.3304	2.0747
Proportion of High RRT Area	3.44%	2.23%	4.24%	1.83%	3.69%	7.73%	3.68%

**Table 3:** Recirculation Fraction Statistics.

	CASE a −90°	CASE b −60°	CASE c −30°	CASE d 0°	CASE e 30°	CASE f 60°	CASE g 90°
The number of particles frozen at the TV	3142	3101	3018	3699	3803	4234	4057
The number of particles frozen at the IVCD	864	939	744	532	434	226	355
Recirculation fraction (%)	21.57%	23.24%	19.78%	12.57%	10.24%	5.07%	8.05%

## Data Availability

Data is contained within the article.
